# Risk Factors for Prescription Opioid Misuse in Older Adults: Findings from the National Survey on Drug Use and Health

**DOI:** 10.1093/geroni/igaf122.2514

**Published:** 2025-12-31

**Authors:** Andie MacNeil, David Burnes

**Affiliations:** University of Toronto, Toronto, Ontario, Canada; University of Toronto, Toronto, Ontario, Canada

## Abstract

Prescription opioid misuse is recognized as a significant public health concern and social problem because it significantly increases the risk of both fatal and non-fatal overdose. Although a large body of research has examined risk factors for prescription opioid misuse, this research has disproportionately focused on younger adults, even though older adults are more likely to have an opioid prescription than any other age cohort. To address this gap in the literature, this study analyzed nationally representative data from the 2023 National Survey on Drug Use and Health to identify risk factors for past-year prescription opioid misuse among older adults (n = 9,762). Stepwise binary logistic regression analyses were conducted to examine risk factors for prescription opioid misuse. In the first model, which only accounted for sociodemographics, risk factors for opioid misuse were being divorced (OR = 1.60; 95% CI: 1.15-2.22) and having an annual income less than $20,000 (OR = 1.55; 95% CI: 1.01-2.39). When physical health factors were added to the model, income was no longer associated with misuse and poor self-rated health was found to be associated with opioid misuse (OR = 1.94; 95% CI: 1.44-2.63). When mental health and other substance use factors were added in the final model, self-rated health remained significantly associated with misuse (OR = 1.70; 95% CI: 1.24-2.34) and a lifetime history of depression was also found to be associated with misuse (OR = 2.06; 95% CI: 1.47-2.88). Findings from this study help inform our basic knowledge on risk factors for prescription opioid misuse, which can help inform targeted screening and intervention.

